# Die zeitlich-räumliche Verteilung von COVID-19 in Köln und beeinflussende soziale Faktoren im Zeitraum Februar 2020 bis Oktober 2021

**DOI:** 10.1007/s00103-022-03573-4

**Published:** 2022-08-03

**Authors:** Florian Neuhann, Sebastian Ginzel, Michael Buess, Anna Wolff, Sabine Kugler, Günter Schlanstedt, Annelene Kossow, Johannes Nießen, Stefan Rüping

**Affiliations:** 1 Gesundheitsamt der Stadt Köln, Neumarkt 15–21, 50667 Köln, Deutschland; 2 Heidelberg Institute of Global Health, Heidelberg University Hospital, Im Neuenheimer Feld 130.3, 69120 Heidelberg, Deutschland; 3 School of Medicine, Levy Mwanawasa Medical University, Lusaka, Sambia; 4 Fraunhofer Institut für Intelligente Analyse und Informationssysteme IAIS, Sankt Augustin, Deutschland; 5 Dezernat für Soziales, Gesundheit und Wohnen – Sozialplanung/Sozialberichterstattung der Stadt Köln, Köln, Deutschland; 6 Institut für Hygiene, Universitätsklinikum Münster, Münster, Deutschland

**Keywords:** Soziale Ungleichheit, Lokale COVID-Eindämmung, Lokale Ausbreitung Köln, Kleinräumige Ausbreitung, Armutsrisiko, Social inequity, Local containment, Local case distribution Cologne, Small scale distribution, Poverty risk

## Abstract

**Hintergrund und Ziele:**

Schon in der frühen Phase der global sehr verschieden verlaufenden COVID-19-Pandemie zeigten sich Hinweise auf den Einfluss sozioökonomischer Faktoren auf die Ausbreitungsdynamik der Erkrankung, die vor allem ab der zweiten Phase (September 2020) Menschen mit geringerem sozioökonomischen Status stärker betraf. Solche Effekte können sich auch innerhalb einer Großstadt zeigen. Die vorliegende Studie visualisiert und untersucht die zeitlich-räumliche Verbreitung aller in Köln gemeldeten COVID-19-Fälle (Februar 2020–Oktober 2021) auf Stadtteilebene und deren mögliche Assoziation mit sozioökonomischen Faktoren.

**Methoden:**

Pseudonymisierte Daten aller in Köln gemeldeten COVID-19-Fälle wurden geocodiert, deren Verteilung altersstandardisiert auf Stadtteilebene über 4 Zeiträume kartiert und mit der Verteilung von sozialen Faktoren verglichen. Der mögliche Einfluss der ausgewählten Faktoren wird zudem in einer Regressionsanalyse in einem Modell mit Fallzuwachsraten betrachtet.

**Ergebnisse:**

Das kleinräumige lokale Infektionsgeschehen ändert sich im Pandemieverlauf. Stadtteile mit schwächeren sozioökonomischen Indizes weisen über einen großen Teil des pandemischen Verlaufs höhere Inzidenzzahlen auf, wobei eine positive Korrelation zwischen den Armutsrisikofaktoren und der altersstandardisierten Inzidenz besteht. Die Stärke dieser Korrelation ändert sich im zeitlichen Verlauf.

**Schlussfolgerung:**

Die zeitnahe Beobachtung und Analyse der lokalen Ausbreitungsdynamik lassen auch auf der Ebene einer Großstadt die positive Korrelation von nachteiligen sozioökonomischen Faktoren auf die Inzidenzrate von COVID-19 erkennen und können dazu beitragen, lokale Eindämmungsmaßnahmen zielgerecht zu steuern.

**Zusatzmaterial online:**

Zusätzliche Informationen sind in der Online-Version dieses Artikels (10.1007/s00103-022-03573-4) enthalten.

## Abstract (en)

**Background and goals:**

Even in the early phase of the COVID-19 pandemic, which took a very different course globally, there were indications that socio-economic factors influenced the dynamics of disease spread, which from the second phase (September 2020) onwards particularly affected people with a lower socio-economic status. Such effects can also be seen within a large city. The present study visualizes and examines the spatio-temporal spread of all COVID-19 cases reported in Cologne, Germany (February 2020–October 2021) at district level and their possible association with socio-economic factors.

**Methods:**

Pseudonymized data of all COVID-19 cases reported in Cologne were geo-coded and their distribution was mapped in an age-standardized way at district level over four periods and compared with the distribution of social factors. The possible influence of the selected factors was also examined in a regression analysis in a model with case growth rates.

**Results:**

The small-scale local infection process changed during the pandemic. Neighborhoods with weaker socio-economic indices showed higher incidence over a large part of the pandemic course, with a positive correlation between poverty risk factors and age-standardized incidence. The strength of this correlation changed over time.

**Conclusion:**

The timely observation and analysis of the local spread dynamics reveals the positive correlation of disadvantaging socio-economic factors on the incidence rate of COVID-19 at the level of a large city and can help steer local containment measures in a targeted manner.

## Hintergrund

Seit Dezember 2019 haben die SARS-CoV-2-Pandemie und die damit verbundene Erkrankung COVID-19 Menschen auf der ganzen Welt vor große Herausforderungen gestellt [1–3]. Bis Anfang November 2021 wurden weltweit 250 Mio. Erkrankungs- und ca. 5 Mio. Todesfälle registriert [4]. Inzwischen rechnet die Weltgesundheitsorganisation (WHO) mit einer Übersterblichkeit von bis zu 16 Mio. neben anderen weitreichenden Folgen der globalen Pandemie [5].

Im Januar 2020 erreichte das neuartige Coronavirus SARS-CoV‑2 Deutschland mit ersten Fällen in München gefolgt von einem Ausbruch im westdeutschen Landkreis Heinsberg [6, 7]. In Köln wurde der erste Fall einer SARS-CoV-2-Infektion am 28.02.2020 registriert. Seitdem wurden bis zum Stichtag dieser Studie, dem 10.10.2021, 65.358 inzidente Fälle gemeldet (Daten Gesundheitsamt Köln). Während der Hauptübertragungsweg für SARS-CoV‑2 durch respiratorische Aufnahme virushaltiger Partikel in Form von Tröpfchen und Aerosolen bald gesichert war, blieben die Verteilung und das Auftreten von Fällen heterogen. Die Entwicklung der Pandemie zeichnete sich sogar durch erhebliche Unterschiede der zeitlich-räumlichen Ausbreitung auf globaler, regionaler und lokaler Ebene aus.

In der Forschung zur Verbreitung der Viruserkrankung spielte neben viralen Eigenschaften und saisonalen, klimatischen Einflüssen auch früh die Frage nach dem Einfluss sozioökonomischer Faktoren eine Rolle. In einem Scoping-Review identifizierten Wachtler et al. [8] bis einschließlich Mitte Juni 2020 bereits 46 Arbeiten zu dieser Fragestellung, zumeist aus den USA und dem Vereinigten Königreich, aber auch jeweils eine Arbeit aus Italien und Deutschland. Trotz der Heterogenität der analysierten Studien in Bezug auf die Indikatoren für den Sozialstatus wurden, abgesehen von der frühen Phase der Pandemie, mehrheitlich nachteilige Effekte für Personen mit niedrigerem sozioökonomischen Status berichtet. Diese beziehen sich auf Auswirkungen wie Inzidenz, Hospitalisierung oder Mortalität [9, 10].

Ein besseres Verständnis des Einflusses dieser Faktoren auf die Ausbreitungsdynamik ist für die Eindämmungs- und Kontrollstrategien auf verschiedenen Ebenen – national, regional, lokal – von hoher Bedeutung. Auch auf städtischer Ebene in Köln unterscheiden sich Stadtviertel und Sozialräume teilweise deutlich in Bezug auf sozioökonomische Faktoren wie Einkommen, Arbeitslosigkeit, Anteil der Personen mit Migrationsgeschichte [11]. Für die optimierte Umsetzung und Steuerung der Eindämmungsstrategie auf der lokalen Ebene einer Großstadt sind die Kenntnis über die zeitlich-räumliche Verteilung der Fälle sowie die zeitnahe Visualisierung der Ausbreitung hinsichtlich des möglichen Einflusses solcher Faktoren sehr nützlich. Vergleichbar wurde in Köln bereits in Bezug auf die Verteilung von Tuberkuloseerkrankungsfällen vorgegangen, auch wenn sich die Verbreitungsdynamik deutlich von der des SARS-CoV‑2 unterscheidet [12].

Das Ziel der hier vorgelegten Untersuchung ist die Darstellung der zeitlich-räumlichen Verteilung gemeldeter COVID-19-Fälle auf der Ebene der Stadtviertel über den lokalen Verlauf der Pandemie bis Oktober 2021 und deren Assoziation zu ausgewählten soziodemografischen und sozioökonomischen Faktoren.

## Methoden

### Setting.

Köln ist die viertgrößte Stadt Deutschlands mit insgesamt 1.083.498 Einwohnern im Jahr 2020 mit einem Anteil von mehr als 30 % im Ausland geborenen Bürgern. Die Stadt ist administrativ in 9 Stadtbezirke und 86 Stadtteile unterteilt. Dies sind die kleinsten Verwaltungseinheiten, für die soziodemografische und sozioökonomische Daten vonseiten der Stadt verfügbar sind (siehe Onlinematerial zu diesem Beitrag, Abb. Z1; Karte von Köln mit Stadtbezirken, Stadtteilen und Einwohnerzahlen).

Das kommunale Gesundheitsamt der Stadt ist gemäß §§ 6,7 IfSG für Meldung, Ermittlung und Einleitung von Maßnahmen zum Schutz vor Weiterverbreitung von meldepflichtigen Infektionserkrankungen, zu denen COVID-19 zählt, zuständig.

Die nach Infektionsschutzgesetz (IfSG; [13]) erfassten Daten aller in Köln gemeldeten Fälle wurden auf Ebene der Stadtviertel geocodiert und kartiert, um potenzielle Hotspots oder Ausbrüche zu erkennen, potenzielle Treiber und Muster der lokalen Pandemie zu identifizieren und schließlich die zeitlich-räumliche Verteilung der gemeldeten Fälle zu visualisieren. Aus den Analyseergebnissen können mögliche Anpassungen der lokalen Kontrollmaßnahmen im Rahmen der übergeordneten Strategien und Vorgaben aus Landes- und Bundesebene abgeleitet werden. Darüber hinaus stehen die Falldaten der Öffentlichkeit in Form einer interaktiven Stadtkarte mit wöchentlichem Update zur Verfügung und unterstützen so die transparente Kommunikation über die epidemische Situation in der Stadt.

Die Bezugseinheit für diese Studie bilden die Stadtbezirke und die dazugehörigen Stadtteile Kölns, für die Daten von soziodemografischen und sozioökonomischen Variablen verfügbar sind [14].

Im Zuge des Ausbruchs entwickelte die Stadt Köln eine Software („Digitales Kontaktmanagement DiKoMa“) zur Dokumentation und zum Fallmanagement [15]. Relevante und meldepflichtige Informationen gemäß den Vorgaben des IfSG aller gemeldeten COVID-19-Fälle und deren Kontaktpersonen werden in DiKoMa erfasst.

### Datengrundlage.

Die Studie basiert auf den in DiKoMa verwalteten Meldedaten aller Indexfälle, die im Rahmen des IfSG verpflichtend im Zeitraum zwischen Februar 2020 und dem 10.10.2021 erhoben und an das Gesundheitsamt gemeldet wurden. Es wurden nur Personen berücksichtigt, die zum Zeitpunkt der Infektion in Köln gemeldet waren oder ihren dauerhaften Aufenthalt in Köln hatten (65.532 COVID-19-Fälle und 137.115 Kontaktpersonen).

In dieser Studie wird die Melderate als Proxyindikator für die Inzidenz verwendet und entsprechend von „kumulierter Inzidenz“ gesprochen.

### Sozioökonomische Indikatoren.

Die Auswahl der Indikatoren orientierte sich an den Ergebnissen des aktuellen Scoping-Reviews zu sozioökonomischer Ungleichheit und COVID-19 [8]. Als Proxyindikatoren für den sozioökonomischen Status wurden Faktoren nach den Kriterien Relevanz und Verfügbarkeit auf Stadtteilebene, orientiert an den Kriterien für die Definition eines Sozialraums mit Förderbedarf in Köln [11], ausgewählt: Anteil der Bevölkerung mit Migrationshintergrund gemäß den in Deutschland verwendeten Standarddefinitionen, Anteil der als arbeitslos registrierten Personen, Anteil der Bedarfsgemeinschaften im Sinne des Zweiten Buches Sozialgesetzbuch (SGB II), die Anteile der Altersverteilung in den 3 Altersgruppen unter 18, 19 bis 64 und über 65 sowie die durchschnittliche Anzahl der Bewohner pro Adresse und die Höhe des Mietspiegels (Tab. 1). Diese Kennzahlen unterliegen einer Multikollinearität, bei der 2 oder mehr dieser Faktoren miteinander korrelieren. Um den Einfluss wechselseitiger Abhängigkeiten (Multikollinearität) zu reduzieren, wurden ein Sozialindex und ein Abhängigkeitsquotient konstruiert. In beide Kennzahlen fließen Risiken für die sozioökonomische Benachteiligung ein.

**Table 1:** 

	*Indikator*	*Quelle und Beschreibung*	*Stadtteil Statistiken*			
			Min	Max	Durchschnitt (Median)	σ
(a)	Arbeitslosenquote (%)	Stadt Köln (31.12.2020)	2,10	24,00	9,49 (8,50)	4,39
(b)	Anteil der Bedarfsgemeinschaften im Sinne des Zweiten Buches Sozialgesetzbuch (SGB II; %)	Stadt Köln (31.12.2020)	0,60	20,20	6,28 (5,05)	3,91
(c)	Migrationsanteil (%)	Stadt Köln (31.12.2020)	20,20	85,10	41,58 (36,95)	14,66
(d)	Sozialindex	$\left(\mathrm{a}\right)+\left(\mathrm{b}\right)+(\mathrm{c})$	25,60	129,30	57,36 (49,80)	22,32
(e)	Mietspiegel (€/m^2^)	Mietspiegel Köln (Q3/2018 [16])	8,60	12,90	9,90 (9,35)	1,20
(f)	Anzahl Personen pro Adresse	Stadt Köln (2019)	3,10	26,64	8,06 (6,61)	4,15
(g)	Bevölkerungsanteil im erwerbsfähigen Alter (19–64 Jahre; %)	Stadt Köln (31.12.2020)	54,10	78,20	63,95 (63,00)	5,20
(h)	Anteil Bevölkerung unter 18 (%)	Stadt Köln (31.12.2020)	7,20	26,80	17,26 (17,25)	3,38
(i)	Anteil Bevölkerung über 65 (%)	Stadt Köln (31.12.2020)	11,30	29,30	18,79 (18,85)	3,93
(j)	Abhängigkeitsquotient	$\frac{\left(\mathrm{g}\right)}{\left(\mathrm{h}\right)+\left(\mathrm{i}\right)}$	27,88	84,84	57,37 (58,73)	12,30
(k)	Einwohnerzahl	Stadt Köln (31.12.2020)	1119	42.634	12.651 (10.827)	9005

*Min* Minimum, *Max* Maximum,* σ* Standardabweichung

Der Sozialindex eines Stadtteils setzt sich aus der Summe des Anteils der Bevölkerung mit Migrationshintergrund, des Arbeitslosenanteils sowie des Anteils der Bedarfsgemeinschaften nach SGB II zusammen. Der Abhängigkeitsquotient errechnet sich aus dem Verhältnis des Anteils der üblicherweise nicht erwerbstätigen Altersgruppen (unter 18 oder über 65 Jahre) und dem Anteil der erwerbsfähigen Altersgruppe (19 bis 64 Jahre).

Da die Einwohnerzahl zwischen den 86 Stadtteilen stark variiert, wurden die Stadtteile mit 3‑stufigen Schwellenwerten für die sozioökonomischen Merkmale gruppiert. Die Schwellenwerte werden so gewählt, dass die Einwohnersummen zwischen den Gruppen ausgeglichen sind (s. Onlinematerial, Tab. Z1). Sozioökonomische Daten wurden aus öffentlich zugänglichen Quellen abgerufen (Tab. 1). Bezugsgröße für die sozioökonomischen Daten ist die Stadtteilebene, eine Analyse in Bezug auf Haushalte oder Einzelpersonen wird nicht durchgeführt.

### Datenanalyse.

Für die zeitlich-räumliche Analyse wurden alle vom Gesundheitsamt erfassten und an das Landeszentrum für Gesundheit gemeldeten Fälle im Zuständigkeitsbereich der Stadt Köln eingeschlossen. Die zeitliche Analyse folgte dem erkennbaren wellenartigen Verlauf der Pandemie: Phase 1 umfasst den Zeitraum von Ende Februar (erste Meldung) bis Juni 2020, Phase 2 den Zeitraum von Juli 2020 bis Februar 2021, Phase 3 den Zeitraum von März 2021 bis Ende Mai 2021 und Phase 4 den Zeitraum von Juni bis 10.10.2021. Der 10.10. wurde als Stichtag gewählt, weil an diesem Tag vorrübergehend die Möglichkeit zur unbezahlten Testung ausgesetzt wurde mit möglichen Auswirkungen auf das Testverhalten. Die Inzidenz in unterschiedlichen Altersgruppen war während des Pandemieverlaufs unterschiedlich stark ausgeprägt. Gleichzeitig werden sozioökonomische Faktoren auch durch die Altersstruktur bestimmt (z. B. Anzahl der Einwohner pro Haushalt). Um die ungleiche Altersverteilung in den Stadtteilen zu berücksichtigen, wurde für alle Analysen eine Altersstandardisierung vorgenommen. Berechnet wurde diese für die einzelnen Stadtteile auf Basis der Altersverteilung in den Altersgruppen: unter 18, 18 bis 29, 30 bis 64, 65 bis 79 und über 80 Jahre.

Zusätzlich zur Entwicklung der Infektionszahlen in den Pandemiephasen wurden jeweils die Zeiträume betrachtet, während derer ein Zuwachs an gemeldeten Fällen um mindestens 2000 Fälle eintrat (Fallzuwachsintervalle). Die Analyse von Fallzuwachsintervallen ermöglicht einen Blick auf das Infektionsgeschehen, der unabhängig von vorher festgelegten Zeiträumen mit irregulären Phasen der Infektionszunahme und -abnahme ist. Das Maß von mindestens 2000 Fällen für ein „Fallzuwachsintervall“ wurde retrospektiv festgelegt, weil es eine Abbildung von Phasen mit niedrigeren Fallmeldungen und solchen mit höheren Werten ermöglicht (minimales Intervall: 5 Tage, maximales Intervall: 150 Tage).

Um den Zusammenhang zwischen einzelnen sozioökonomischen Faktoren und der altersstandardisierten Inzidenz während der Pandemiephasen zu beschreiben, wurden die sozioökonomischen Faktoren in Intervalle unterteilt. Die Einteilung wurde so gewählt, dass die Stadtteile je Intervall in der Summe vergleichbar viele Einwohner hatten (s. Onlinematerial, Tab. Z1).

### Statistische Auswertung.

Die Unterschiede der Inzidenzen über die oben beschriebenen Zeiträume zwischen den Intervallen der sozioökonomischen Indikatoren wurden auf Basis eines Kruskal-Wallis-Tests und eines Dunns-Post-hoc-Tests ermittelt. Als Signifikanzniveau wurde α = 0,05 festgelegt.

Zusätzlich wurde eine Regressionsanalyse durchgeführt, um die Gewichtung mehrerer sozioökonomischer Kennzahlen und die Inzidenz als unabhängige Variable in den Fallzuwachsintervallen systematisch zu beschreiben. Hierbei wurde je ein unabhängiges generalisiertes lineares Modell (GLM) für jedes Fallzuwachsintervall gebildet. Als Signifikanzniveau für die Gewichte der Modelle wurde α = 0,05 festgelegt und die *p*-Werte entsprechend der Anzahl der gebildeten Modelle Bonferroni-korrigiert, siehe Ergebnisse zur kombinierten Einflussstärke der sozioökonomischen Faktoren. Unabhängige Variablen wurden skaliert und standardisiert, die abhängige Variable log-transformiert. Als Maß für die Multikollinearität wurde für die hier untersuchten Einflussfaktoren der Variance Inflation Factor (VIF) bestimmt. Dieser beschreibt, wie gut eine unabhängige Variable von den anderen unabhängigen Variablen beschrieben wird, wobei VIF-Werte größer als 5 auf Kollinearität hinweisen. Die Analysen wurden mit der Software „R“ („R“ Statistical Computing, Wien, Österreich) Version 4.1 durchgeführt, die Visualisierung wurde auf Basis der Grammar of Graphics Bibliothek ggplot2 [17] Version 3.3.6 und rstatix [18] Version 0.7.0 erstellt.

### Ethische Aspekte.

Alle verwendeten Daten wurden im rechtlichen Rahmen des IfSG erhoben. Daher wurde eine individuelle Einwilligung als nicht erforderlich angesehen. Die räumliche Auswertung war auf die Ebene der Stadtviertel begrenzt und nicht auf Haushalte oder Individuen. Alle Daten werden in einer sicheren Datenbank auf einem Server in der Stadtverwaltung gespeichert. Die Datenbankprozesse und die Speicherung wurden von den Datenschutzbeauftragten der Stadt bewertet und gemäß den Vorgaben der Datenschutzgrundverordnung und des Bundesdatenschutzgesetzes gebilligt. Die analysierenden Partnerinstitutionen schlossen zusätzlich eine Vereinbarung zum Datenschutz ab. Die zu analysierenden Daten wurden vor der Übertragung vom kommunalen Server an die Partnerinstitution anonymisiert.

## Ergebnisse

**Figure 1:**
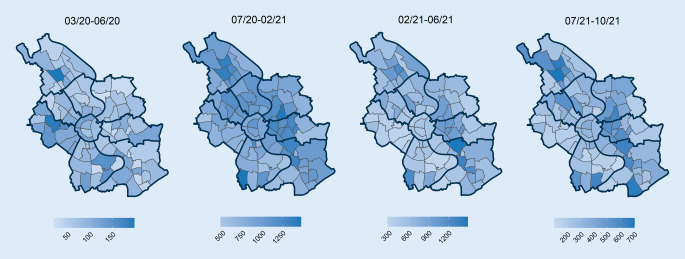

Die epidemiologischen Kennzahlen für den gewählten Zeitraum sind in Tab. 2 dargestellt, die kumulative epidemiologische Situation in Abb. 1.

**Table 2:** 

Beobachtungszeitraum	01.03.2020 bis 10.10.2021 (589 Tage)	
Gesamtzahl gemeldeter Fälle und Kontaktpersonen	209.353	
Registrierte Erkrankungsfälle (PCR-positive Indexfälle)	65.358	
Geschlechtsverteilung (Indexfälle)	Divers	8 (0,01 %)
	Weiblich	33.331 (51 %)
	Männlich	31.996 (49 %)
Altersmedian (Indexfälle)	36 (IQA: 29)	
Mittlere Anzahl von gemeldeten Kontaktpersonen	2,09 (IQA: 3)	

*IQA* Interquartilabstand, *PCR* Polymerase-Kettenreaktion („polymerase chain reaction“)

### Sozioökonomische Faktoren.

**Figure 2:**
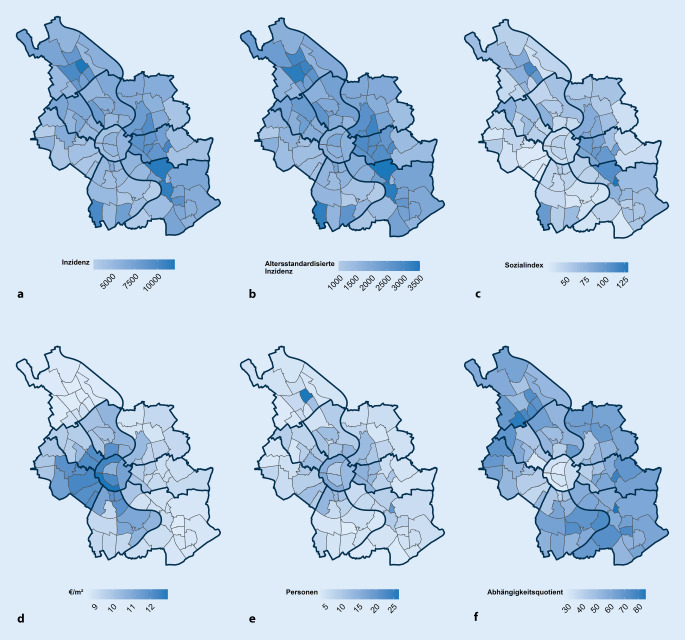

**Figure 3:**
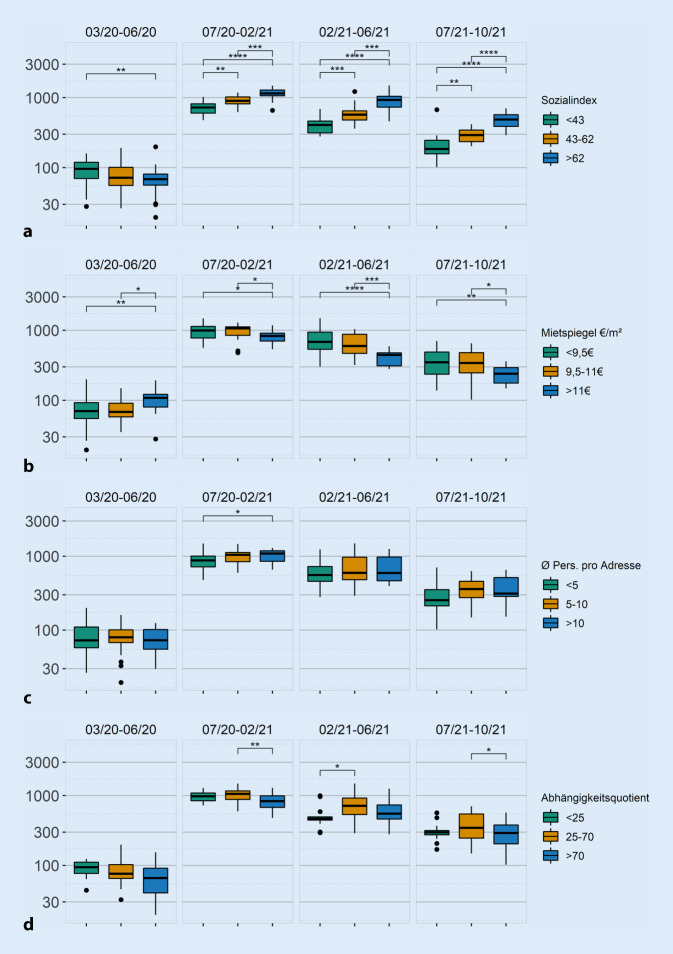

Abb. 2 stellt die räumliche Verteilung der kumulierten Inzidenzen in Bezug zu ausgewählten sozioökonomischen Variablen. Bereits visuell lässt sich erkennen, dass die kumulierte Inzidenz (a), die altersstandardisierte Inzidenz (b) und der gebildete Sozialindex (c) eine ähnliche Verteilung über die Stadtteile zeigen, während die Höhe des Mietspiegels ein anderes Verteilungsmuster aufweist. Für die anderen Faktoren lässt sich optisch kein klares Muster erkennen. Die Ergebnisse über die gewählten 4 Phasen werden in Abb. 3a–d dargestellt.

### Kombinierte Einflussstärke der sozioökonomischen Faktoren.

**Figure 4:**
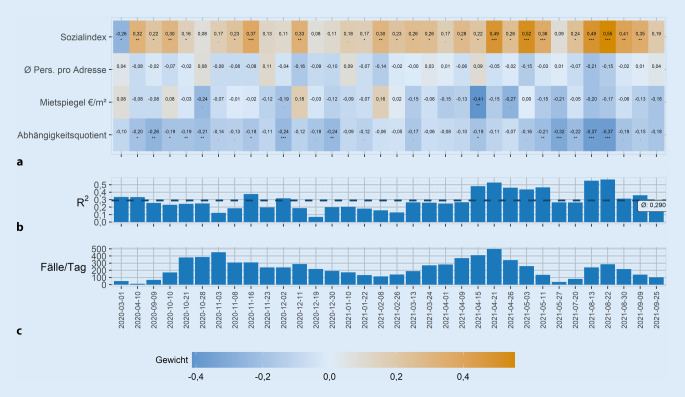

Die Einflussstärke der sozioökonomischen Faktoren wurde in 34 Fallzuwachsintervallen analysiert (Abb. 4). Der VIF-Wert liegt für alle Einflussfaktoren unter 4, sodass der Effekt von Multikollinearität gering ausfällt. Der Anteil der erklärten Varianz durch die Modelle schwankt je nach Intervall und liegt zwischen 6,9 % im Zeitraum 19. bis 29.12.2020 und 57 % im Zeitraum 22. bis 29.08.2021, bei einem Durchschnittswert von 29 %. Die Modelle wurden unabhängig voneinander gebildet, zeigen aber, dass während 24 der 34 Fallzuwachsintervallen der Sozialindex einen signifikanten Beitrag zur Vorhersage der stadtteilspezifischen Inzidenz leistet. In 23 dieser Intervalle ist sein Beitrag dabei positiv, d. h., eine höhere sozioökonomische Belastung der Stadtteile führt zu einer höheren Inzidenz. Umgekehrt trägt der Abhängigkeitsquotient in 14 Fallzuwachsintervallen signifikant negativ zur Vorhersage bei. In einem Intervall zeigt der Mietspiegel der Stadtteile eine negative Assoziation mit der Inzidenz. Die durchschnittliche Anzahl an einer Adresse gemeldeter Personen trägt in keinem Fallzuwachsintervall zur Vorhersage der Inzidenz bei.

Für 11 Fallzuwachsintervalle erklären die Modelle mehr als 33 % der Varianz. Für die 10 Modelle mit positivem Sozialindexgewicht ist ein Sozialindexpunkt mit einem Inzidenzzuwachs von 2 % (Median) assoziiert (25 %-Quartil: 1,75, 75 %-Quartil: 2,86). Für alle Modelle mit hoher erklärter Varianz geht der Abhängigkeitsindex negativ in die Vorhersage ein, wobei ein Zuwachs von einem Abhängigkeitspunkt mit einem Medianabfall um 1,3 % der Inzidenz verbunden ist (25 %-Quartil: 0,98, 75 %-Quartil: 1,5). Die stärkste Inzidenzreduktion je Einheit ist für den Mietspiegel pro m^2^ zu beobachten. Für das Intervall vom 15. bis 21.04.2021 werden in Stadtteilen, in denen der Mietspiegel um einen Euro höher liegt, 28 % weniger Fälle vorhergesagt (s. Onlinematerial, Abb. Z2).

## Diskussion

Die vorliegende Untersuchung beruht auf der Auswertung aller gemeldeten COVID-19-Fälle in Köln bis zum 10.10.2021 und verfolgt das Ziel, die kleinräumige Ausbreitungsdynamik in einer Großstadt besser zu verstehen. Die Analyse wurde im Rahmen eines Kooperationsprojektes zwischen dem Gesundheitsamt der Stadt Köln und der Fraunhofer Gesellschaft als operative Forschung in einer Phase maximaler Arbeitsbelastung seitens des Gesundheitsamtes durchgeführt.

Die Analyse des räumlich-zeitlichen Verlaufs der COVID-19-Pandemie auf der Ebene der Stadtteile in der viertgrößten Stadt Deutschlands zeigt zunächst einmal eine geografische Verschiebung von südwestlichen zu nordöstlichen Teilen der Stadt innerhalb von 19 Monaten. Diese Ausbreitung korreliert mit soziodemografischen und sozioökonomischen Faktoren auf Stadtteilebene. Ausgehend von Stadtteilen mit niedrigem Armutsrisiko verschiebt sich nach den ersten 3 Monaten die Häufigkeit der gemeldeten Fälle und zeigt danach höhere Inzidenzen in Stadtteilen mit niedrigen sozioökonomischen Charakteristika und somit erhöhten Armutsrisiken.

Zeitlich fällt der Beginn der Pandemie in Köln mit dem Ende der Wintersaison und der Karnevalssaison zusammen. Eine höhere Reiseaktivität (Wintersport) oder die Teilnahme an Karnevalsitzungen von sozial besser gestellten Personen mit Wohnsitz in entsprechenden Stadtteilen können zur Erklärung der dort initial höheren Inzidenz herangezogen werden. Der weitere Verlauf der lokalen Epidemie zeigt dann höhere Inzidenzen unter der Bevölkerung in ärmeren Stadtteilen.

Für die Analyse wurden keine Daten auf Haushaltsebene oder individueller Ebene zu sozioökonomischen Variablen herangezogen. Wir konstruierten auf der Basis von Sozialdaten der Stadt auf Stadtteilebene einen ungewichteten Sozialindex als Summe aus Arbeitslosenquote (%), Anteil der Bedarfsgemeinschaften nach SGB II (%) und Migrationsanteil (%). Auch wenn dieser nicht die Komplexität des German Index multipler Deprivation abbildet, erklärt der Index während langer Phasen der Pandemie im Schnitt mit 29 % einen beträchtlichen Anteil der Varianz (Abb. 4; [19]). Dabei vermeidet der summierte Index gleichzeitig die kausale Attribuierung zu einem der 3 eingehenden Faktoren, welche mit der vorliegenden Untersuchung nicht geleistet werden soll und kann.

Der Anteil der erklärten Varianz schwankt im Verlauf des Beobachtungszeitraums. Diese Schwankung im Anteil der erklärten Varianz zeigt, dass die beschriebenen sozioökonomischen Faktoren die Inzidenz über die Zeit unterschiedlich gut abbilden. So fällt dieser Anteil in einer Phase vor Weihnachten 2020 und im Februar 2021 deutlich unter den Mittelwert, während er in der Hochphase der 3. Welle im April wieder deutlich ansteigt. Das heißt, dass zu verschiedenen Zeiten und im Zusammenhang mit Fallzahlen die Bedeutung des Sozialindex für die Infektionsdynamik Schwankungen unterliegt, die durch unsere Studie nicht erklärt werden. Faktoren wie die Witterung, Maßnahmen wie der Lockdown und Testangebote können zu Verhaltensänderungen in verschiedenen Bevölkerungsstrata beitragen, sodass der Einfluss der sozioökonomischen Variablen geringer ausfällt. Ähnliche Veränderungen über die Zeit werden auch in anderen Studien berichtet. Plümper and Neumayer [20] stellen die Unterschiede zwischen der ersten und zweiten Pandemiephase in Deutschland heraus, vom anfänglichen Eintrag und der Verbreitung in eher wohlhabenderen Gruppen im Gegensatz zu späteren Phasen der Pandemie, und führen an, dass im weiteren Verlauf die Möglichkeit, Kontakte zu reduzieren – z. B. durch Homeoffice – wichtig für die Vermeidung von Infektionen und diese Möglichkeit für ärmere Bevölkerungsgruppen geringer ist. Ähnliche Beobachtungen machen auch Wachtler et al. basierend auf den Meldedaten zu SARS-CoV‑2 bis Juni 2020 [21]. Padellini et al. zeigen für den Zeitraum Juni 2020 bis September 2021 für das Vereinigte Königreich ebenfalls eine variierende Ausprägung des Einflusses durch die ethnische Komposition und des dort verwendeten Deprivationsindexes auf Inzidenz und Prävalenz [22].

Auch wenn weitere, von uns nicht aufgedeckte Faktoren zur lokalen Verbreitung und Infektionsdynamik beitragen, zeigt sich über die verschiedenen Phasen der Pandemie und der Kontrollmaßnahmen ein beträchtlicher Einfluss sozioökonomischer Faktoren.

Die Betrachtung der Inzidenzentwicklung innerhalb der Fallzuwachsintervalle zeigt, dass die Entwicklung innerhalb der bisherigen wellenförmig verlaufenden Infektionszyklen unterschiedlichen Dynamiken folgt. Phasenweise können sozioökonomische Faktoren ein guter Indikator für die Vorhersage des Infektionsgeschehens sein. So ist im Großteil der Fallzuwachsintervalle (70,5 %) der Sozialindex eine signifikante Kenngröße zur Vorhersage der Inzidenz. Intervalle, in denen dieser Index keinen signifikanten Beitrag zu den Modellen liefert, sind dadurch gekennzeichnet, dass andere sozioökonomische Kenngrößen signifikante Beiträge leisten. Man erkennt auch, dass Effekte von Verhaltensänderung innerhalb der Bevölkerung, zum Beispiel zur Weihnachtszeit, dazu führen, dass Vorhersagen allein auf Basis der o. g. sozioökonomischen Faktoren nur ein unvollständiges Bild generieren. Die Betrachtung von Fallzuwachsintervallen als unabhängig voneinander bedarf weiterer Untersuchungen. Zwar weisen die einfach interpretierbaren linearen Modelle eine hohe Modellgüte gemessen am Anteil der erklärten Varianz auf (durchschnittlich 29 % und phasenweise bis zu 57 %), gleichzeitig unterliegen diese Schwankungen, die zusätzliche äußere Effekte vermuten lassen.

Obwohl die Art der Übertragung des Virus für alle Menschen gleich ist, legen die Ergebnisse eine Abhängigkeit der COVID-19-Inzidenz von sozioökonomischen Faktoren bzw. sozialen Determinanten der Gesundheit nahe, ähnlich wie bei anderen Infektionskrankheiten, z. B. der Tuberkulose. Strukturelle Faktoren wie Wohnraum und arbeitsbedingte Faktoren können neben Verhaltensfaktoren eine Rolle spielen und erscheinen angesichts des Übertragungsmusters von SARS-CoV‑2 plausibel. Allerdings scheint die Interaktion komplex zu sein, wie durch die unterschiedlichen Einflüsse einzelner Faktoren in verschiedenen Intervallen verdeutlicht wird. Unsere Daten erlauben es jedoch nicht, Rückschlüsse auf kausale Zusammenhänge zu ziehen. Sie stimmen aber überein mit Daten und Beobachtungen aus anderen Ländern und Umgebungen, die ebenfalls einen Zusammenhang zwischen den Indizes für soziale Benachteiligung und der Inzidenz und Schwere des Verlaufs von COVID-19 zeigen [9, 10, 23]. Die hier präsentierten Daten aus Köln reflektieren ebenso eine stärkere Korrelation des Sozialindexes auf die Inzidenz zum Jahresende 2020 und zu Beginn von 2021 wie die von Hoebel et al. in Bezug auf die deutschlandweiten Fallzahlen [24]. Auch Ehlert zeigt in differenzierten Modellen einen Zusammenhang von sozioökonomischen Faktoren und COVID-19 in Deutschland während der ersten Welle auf [25].

### Stärken und Grenzen dieser Studie

Die Stärke der vorliegenden Studie liegt im vollständigen Einschluss aller in Köln in der Zeit von Februar 2020 bis Oktober 2021 gemeldeten Fälle. Dies ist mangels einer großen Bevölkerungskohorte die beste Annäherung an die tatsächliche Fallverteilung. Eine solche Kohortenstudie wurde in Köln durchgeführt und könnte weitere Aufschlüsse zur Inzidenz generieren [26]. Zum jetzigen Zeitpunkt lässt sich eine Verzerrung bei der Erkennung und dem Ausmaß der Untererfassung von Fällen, z. B. aufgrund von asymptomatischen Verläufen, Verfügbarkeit von Tests, Teststrategie und Testverhalten in der Bevölkerung, nicht ausschließen. Diese Verzerrung führt u. E. aber eher zu einer Unterschätzung des Beitrags der gewählten sozioökonomischen Faktoren, z. B. durch den schwierigeren Zugang zur Testung bei sprachlichen oder kulturellen Barrieren. Aufgrund der Erfassung und Auswertung der Fälle bis einschließlich Oktober 2021 ist die Verteilung während der vorherrschenden Ausbreitung bis zur Deltavariante des Virus erfasst. Zu lokalen Verbreitungsmustern bei Vorherrschen der höher kontagiösen Omikronvariante und deren Subvarianten kann hingegen zum jetzigen Zeitpunkt keine Aussage getroffen werden. Hohe Kontagiosität könnte zu einer gleichförmigeren Ausbreitung in verschiedenen Bevölkerungsgruppen führen. Allerdings zeigen die Daten des Robert Koch-Instituts einen Zusammenhang von sozialer Deprivation und Inzidenz auch für die 4. Welle der Pandemie [27].

Die vorliegende Untersuchung war von Anfang an als beschreibende und ökologische Studie gedacht. Ziel war es, die Entwicklung der lokalen Epidemie besser zu verstehen und nicht gezielt die kausalen Zusammenhänge zu untersuchen.

Die Tatsache der Meldung eines Falls in einem Stadtteil ist nicht unbedingt gleichbedeutend mit dem Erwerb der Infektion im gleichen Gebiet. Allerdings legen unveröffentlichte Daten aus DiKoMa nahe, dass tatsächlich ein Großteil der gemeldeten Infektionen im gleichen Stadtteil erworben wird.

Darüber hinaus befasst sich die Studie nur mit den Fallmeldungen und enthält keine stadtteilbezogenen Outcome-Daten zu Krankenhausaufenthalten, Krankheitsverlauf oder Mortalität. Auch hier kann der ökologische Charakter unserer Daten gezielte Studien anregen.

Die Ergebnisse der hier vorgestellten Untersuchungen sollten noch durch gezielte Studien bestätigt werden, die sich auf sozioökonomische Treiber der Pandemie konzentrieren, welche auch die Haushaltsebene einbeziehen. Die vorliegende Analyse auf der Stadtteilebene kann jedoch zu Einsichten in die lokale Inzidenz- und Prävalenzlast führen und ermöglicht eine handlungsorientierte Planung. Beispielsweise wurden nach einer ersten Präsentation der Ergebnisse mobile Test- und Impfkampagnen in Stadtbezirken und Stadtteilen mit anhaltend höherer Inzidenz gestartet. Darüber hinaus stehen die Falldaten der Öffentlichkeit in Form einer interaktiven Stadtkarte mit wöchentlichem Update zur Verfügung und unterstützen so die transparente Kommunikation über die epidemische Situation in der Stadt.

## Fazit

Basis für diese Analysen ist die digitalisierte Bündelung von Daten in der von der Stadt Köln entwickelten Datenbank. Deren Darstellung in einem geografischen Informationssystem ermöglicht es, Muster der räumlichen Verteilung von SARS-CoV-2/COVID-19 im Kölner Stadtgebiet im Zeitverlauf zu identifizieren und stadtteilbezogen assoziierte sozioökonomische Faktoren zu erkennen.

Im September 2020 beschrieb R. Horton in einem Kommentar im *Lancet* COVID-19 als „Syndemic“, um darauf hinzuweisen, dass bei COVID-19 biologische und soziale Faktoren interagieren und insbesondere soziale Ungleichheit eine wesentliche Rolle bei der Infektion und beim Verlauf der Erkrankung spielt [28]. Untersuchungen zu anderen Erkrankungen verweisen auch auf eine sozial ungleiche Verteilung von Gesundheitsrisiken, so zeigt sich auf nationaler Ebene ein Zusammenhang der sozialen Deprivation mit Erkrankungen und Risikofaktoren für einen schwerwiegenderen klinischen Verlauf, z. B. bei einem erhöhten Body-Mass-Index (BMI) oder bei einer Hypertonie [29, 30].

Diese Studie zeigt, dass sich der Zusammenhang zwischen der zeitlich-räumlichen Verteilung der SARS-CoV-2-/COVID-19-Inzidenzen und sozioökonomischen Faktoren nicht nur auf Bundes- und Landkreisebene, sondern auch kleinräumig in der Großstadt Köln nachvollziehen lässt. Im Pandemieverlauf kam es hierbei zu einer Inzidenzverlagerung von Stadtteilen mit geringem Armutsrisiko zu Stadtteilen mit höherem Armutsrisiko, gemessen an den hier verwendeten sozioökonomischen Kennzahlen. Die Stärke, mit der diese Faktoren die Inzidenz beeinflussen, unterscheidet sich in Abhängigkeit vom betrachteten Zeitraum teilweise erheblich. Solche kleinräumigen Analysen können helfen das Infektionsgeschehen lokal besser zu verstehen und die Bekämpfungsstrategie anzupassen.

Untersuchungen wie die vorliegende führen zu Public-Health-relevanten Erkenntnissen. Diese können zur Vorbereitung auf eine neue Pandemiewelle oder vergleichbare Ereignisse genutzt werden. Damit erfüllt die epidemiologische Forschung in Gesundheitskrisen eine wichtige Aufgabe [31–34]. Auch wenn keine kausale Beziehung belegt werden kann, unterstützen unsere Ergebnisse in der Zusammenschau mit anderen Studien den Einfluss nachteiliger sozioökonomischer Faktoren auf die Inzidenz von COVID-19, rechtfertigen gezielte Präventionsmaßnahmen und Interventionen für vulnerable Bevölkerungsgruppen und können damit einen Beitrag zur effizienten Pandemiebekämpfung leisten. Umfassende Forschung ist notwendig, um zu verstehen, wie sich das individuelle Infektionsrisiko in Abhängigkeit von sozioökonomischen Faktoren verhält.

## Supplementary Information




