# Supplementary material for: Spatio-temporal distribution of COVID-19 in Cologne and associated socio-economic factors in the period from February 2020 to October 2021
Source: Bundesgesundheitsblatt Gesundheitsforschung Gesundheitsschutz. 2022 Aug 3;65(9):853–62. [Article in German] doi: 10.1007/s00103-022-03573-4 (PMC9362610; doi:10.1007/s00103-022-03573-4)
Supplement: Supplementary file 1 [file 103_2022_3573_MOESM1_ESM.pdf]

Bundesgesundheitsblatt – Gesundheitsforschung – Gesundheitsschutz, Ausgabe 9/2022

Onlinematerial zum Beitrag:

Die zeitlich-räumliche Verteilung von COVID-19 in Köln und beeinflussende soziale Faktoren im Zeitraum Februar 2020 bis Oktober 2021

Florian Neuhann[1,3,4] *, Sebastian Ginzel[2] *,Michael Buess[1] , Anna Wolff[1] , Sabine Kugler[2] ,Günter Schlanstedt[6] , Annelene Kossow[1,5], Johannes Nießen[1], Stefan Rüping[2]

* die Autoren haben gleichermaßen beigetragen

[1] Gesundheitsamt der Stadt Köln, Köln, Deutschland
[2] Fraunhofer Institut für Intelligente Analyse und Informationssysteme IAIS, Sankt Augustin, Deutschland
[3] Heidelberger Institut für Global Health Universitätsklinikum Heidelberg, Heidelberg, Deutschland
[4] School of Medicine Lewy Mwanawasa Medical University, Lusaka, Zambia
[5] Institut für Hygiene, Universitätsklinikum Münster, Münster, Deutschland
[6] Dezernat für Soziales, Gesundheit und Wohnen - Sozialplanung/Sozialberichterstattung der Stadt Köln, Köln, Deutschland

Korrespondenzadresse:
Dr.med. Florian Neuhann
Gesundheitsamt der Stadt Köln
Neumarkt 15-21
50667 Köln
Deutschland
florian.neuhann@uni-heidelberg.de

Inhalt:

Abbildung Z1: Karte der 86 Kölner Stadtteile mir der zugehörigen Einwohnerzahl.

Abbildung Z2: Fallanstieg (in %) pro Punkte-Inkrement je Attribut.

Tabelle Z1: Stadtteilgruppierung nach sozioökonomischen Faktoren.

Abbildung Z1: Karte der 86 Kölner Stadtteile mir der zugehörigen Einwohnerzahl.

Hervorgehoben sind auch die 9 Stadtbezirke, welche die nächstgrößere Gliederungseinheit darstellen. (Eigene Abbildung)

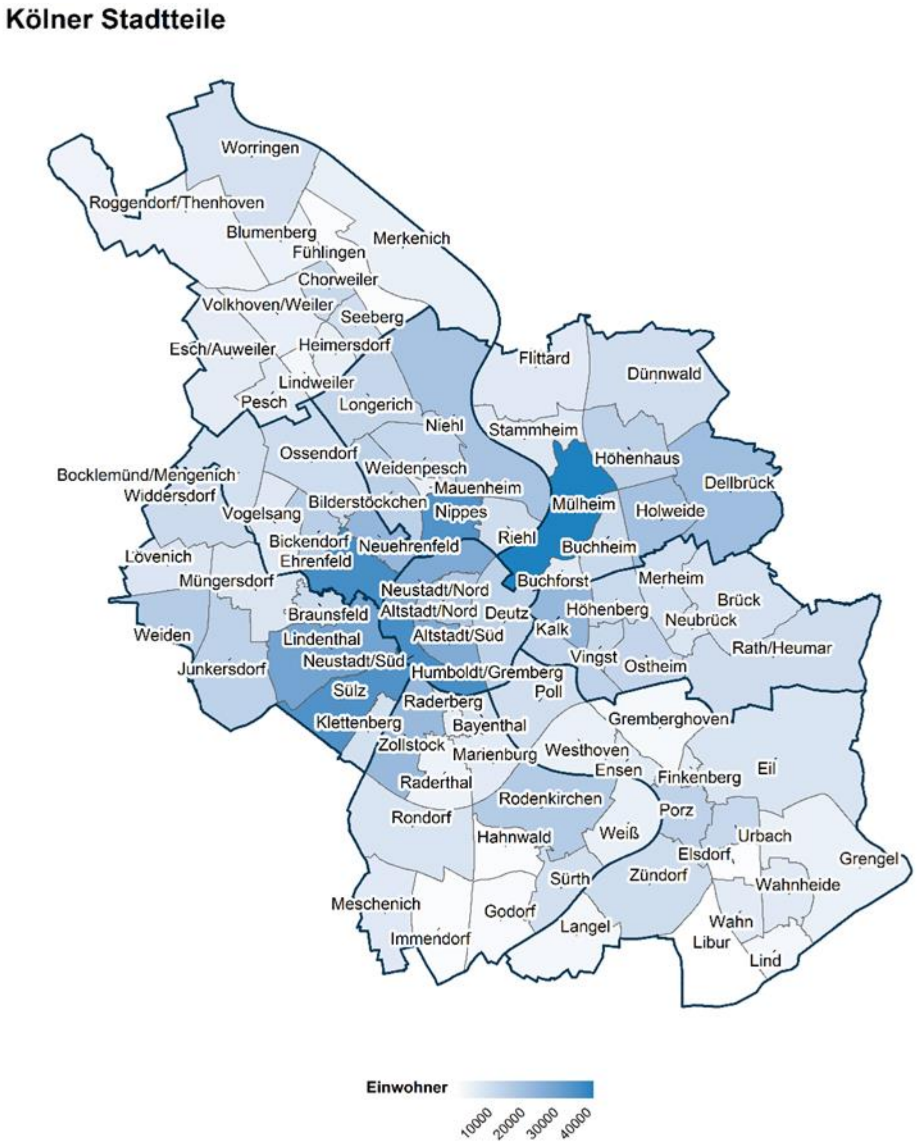

Abbildung Z2: Fallanstieg (in %) pro Punkte-Inkrement je Attribut. (Eigene Abbildung)

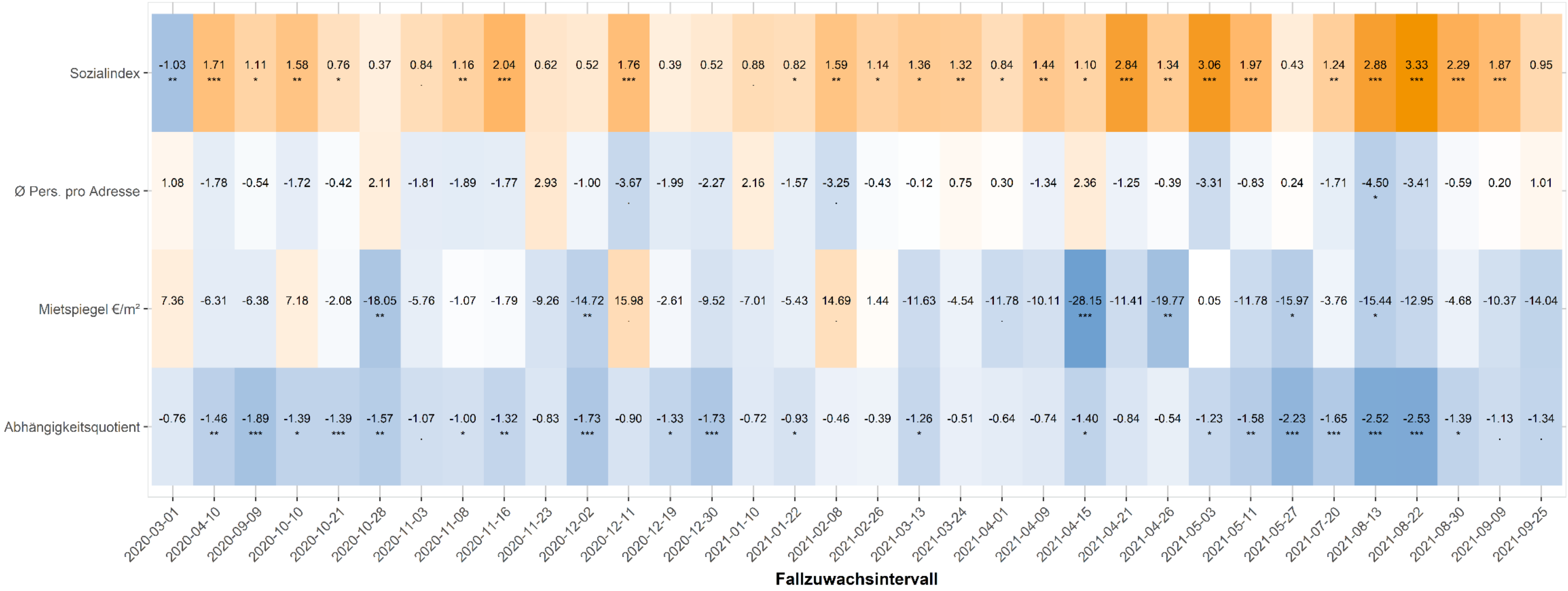

Tabelle Z1: Stadtteilgruppierung nach sozioökonomischen Faktoren. Grenzwerte für Gruppierung und Anzahl Einwohner pro Gruppen sind hier angegeben und dienen als Basis für die Darstellung der Inzidenzverteilung.

**Faktor**	**Einwohner (Anzahl Stadtteile)**
Einwohner (gesamt)	1.088.040 (86)
**Sozialindex**	
<43	350.657 (24)
43-62	364.337 (31)
>62	373.046 (31)
**Ø Pers. pro Adresse**	
<5	362.092 (45)
5-10	415.061 (25)
>10	310.887 (16)
**Mietspiegel (€/sqm)**	
<9.5€	380.954 (45)
9.5-11€	315.853 (22)
>11€	391.233 (19)
**Abhängigkeitsquotient**	
<25	368.820 (13)
25-70	359.026 (35)
>70	360.194 (38)
